# Improvements in Patient Monitoring in the Intensive Care Unit: Survey Study

**DOI:** 10.2196/19091

**Published:** 2020-06-19

**Authors:** Akira-Sebastian Poncette, Lina Mosch, Claudia Spies, Malte Schmieding, Fridtjof Schiefenhövel, Henning Krampe, Felix Balzer

**Affiliations:** 1 Department of Anesthesiology and Intensive Care Medicine Charité – Universitätsmedizin Berlin, Corporate Member of Freie Universität Berlin, Humboldt-Universität zu Berlin, and Berlin Institute of Health Berlin Germany; 2 Einstein Center Digital Future Berlin Germany

**Keywords:** digital health, patient monitoring, monitoring, intensive care medicine, intensive care unit, technological innovation, user-centered, usability, online survey, transdisciplinary, REDCap, email

## Abstract

**Background:**

Due to demographic change and, more recently, coronavirus disease (COVID-19), the importance of modern intensive care units (ICU) is becoming apparent. One of the key components of an ICU is the continuous monitoring of patients' vital parameters. However, existing advances in informatics, signal processing, or engineering that could alleviate the burden on ICUs have not yet been applied. This could be due to the lack of user involvement in research and development.

**Objective:**

This study focused on the satisfaction of ICU staff with current patient monitoring and their suggestions for future improvements. We aimed to identify aspects of monitoring that interrupt patient care, display devices for remote monitoring, use cases for artificial intelligence (AI), and whether ICU staff members are willing to improve their digital literacy or contribute to the improvement of patient monitoring. We further aimed to identify differences in the responses of different professional groups.

**Methods:**

This survey study was performed with ICU staff from 4 ICUs of a German university hospital between November 2019 and January 2020. We developed a web-based 36-item survey questionnaire, by analyzing a preceding qualitative interview study with ICU staff, about the clinical requirements of future patient monitoring. Statistical analyses of questionnaire results included median values with their bootstrapped 95% confidence intervals, and chi-square tests to compare the distributions of item responses of the professional groups.

**Results:**

In total, 86 of the 270 ICU physicians and nurses completed the survey questionnaire. The majority stated they felt confident using the patient monitoring equipment, but that high rates of false-positive alarms and the many sensor cables interrupted patient care. Regarding future improvements, respondents asked for wireless sensors, a reduction in the number of false-positive alarms, and hospital standard operating procedures for alarm management. Responses to the display devices proposed for remote patient monitoring were divided. Most respondents indicated it would be useful for earlier alerting or when they were responsible for multiple wards. AI for ICUs would be useful for early detection of complications and an increased risk of mortality; in addition, the AI could propose guidelines for therapy and diagnostics. Transparency, interoperability, usability, and staff training were essential to promote the use of AI. The majority wanted to learn more about new technologies for the ICU and required more time for learning. Physicians had fewer reservations than nurses about AI-based intelligent alarm management and using mobile phones for remote monitoring.

**Conclusions:**

This survey study of ICU staff revealed key improvements for patient monitoring in intensive care medicine. Hospital providers and medical device manufacturers should focus on reducing false alarms, implementing hospital alarm standard operating procedures, introducing wireless sensors, preparing for the use of AI, and enhancing the digital literacy of ICU staff. Our results may contribute to the user-centered transfer of digital technologies into practice to alleviate challenges in intensive care medicine.

**Trial Registration:**

ClinicalTrials.gov NCT03514173; https://clinicaltrials.gov/ct2/show/NCT03514173

## Introduction

### Background

In the near future, continuous monitoring of patients' vital signs will play an increasingly important role in alleviating the burden on the health care system caused by demographic change and, more recently, coronavirus disease (COVID-19) [1]. Both lead to an increased number of critically ill patients requiring intensive medical care, including mechanical ventilation and patient monitoring. However, existing advances in informatics, signal processing, or engineering have not yet been applied to patient monitoring [2], making it primarily an alarm system notifying health care providers whenever a patient's parameter deviates from preset values that are considered safe. To accelerate technology transfer into clinical routine, it may be beneficial to include users' pain points and suggestions for research and development.

Patient monitoring can be applied across almost all health sectors, which underlines its importance and the potential offered by digitalization. First, patients can monitor themselves preventively (eg, for atrial fibrillation), even with a consumer product such as the Apple Watch [3]. Second, remote monitoring of patients over long distances is a crucial component of telemedicine, which is becoming increasingly widespread in most areas of medicine [4]. Third, patient monitoring might soon be mandatory in general wards due to a shift in inpatient clientele toward the more critically ill [5,6]. Finally, patient monitoring produces high-frequency data that are a valid and essential source for clinical decision support systems (CDSS) based on artificial intelligence (AI), opening up many possibilities for precision medicine [7].

In the intensive care unit (ICU), as one of the most technologically enhanced medical areas, staff have used monitoring technologies over decades. In a previous qualitative study from our research group, ICU staff demanded wireless, noninvasive, and interoperable monitoring sensors and improved alarm management for a future patient monitoring system [8]. Mobile phones were desired as displays for remote patient monitoring, and CDSS based on AI was considered useful. To validate these inclinations in a larger cohort, we designed this survey study of ICU staff.

### Aim

This survey study focuses on ICU staff members’ satisfaction with the current patient monitoring system and their suggestions for future technological improvements. In particular, we aimed to identify the aspects of patient monitoring that disturb patient care, the display devices most appropriate for the ICU for remote patient monitoring on the hospital premises, the use cases for AI in the ICU, and whether ICU staff is willing to improve their digital literacy or contribute to product improvement. With regard to the multiprofessional structure of ICU teams, we further desired to uncover differences in perspectives between different health professions in the ICU.

## Methods

### Ethics Approval and Consent to Participate

The ethical approval for this study was granted by the Ethics Commission of the Charité – Universitätsmedizin Berlin (EA1/031/18). Participation in the survey was voluntary. Prior to the study, all participants provided their written consent.

### Setting

This survey study was performed with ICU staff from 4 ICUs of a German university hospital, between November 2019 and January 2020 as a substudy for the implementation of the virtual patient monitoring platform Vital Sync 2.4 (Medtronic plc). This new system was implemented between May 2018 and June 2019 in one of the 4 ICUs as a secondary patient monitoring system to remotely monitor patients via tablet computers. As the primary patient monitoring system, the Philips IntelliVue patient monitoring system (Koninklijke Philips NV; MX800 software version M.00.03; MMS X2 software version H.15.41-M.00.04) was used in all 4 ICUs at the time of the study. COPRA 6 (COPRA System GmbH) was used as the patient data management system (PDMS).

### Study Design

We chose a cross-sectional survey design, and developed a web-based questionnaire [9,10]. Survey item generation was initiated through the analysis of a preceding qualitative interview study with ICU staff about clinical requirements of future patient monitoring, and was saturated in focus group sessions within the research team [8]. Items were then grouped into topics, and 5 to 6 items per topic were anticipated. We chose a 5-point Likert-type scale as an ordinal response format, with the options “Strongly disagree” (score=1), “Disagree” (score=2), “Undecided” (score=3), “Agree” (score=4), and “Strongly agree” (score=5). In pretests with associated research colleagues, redundant items were eliminated without removing whole topics. Pilot testing was conducted face-to-face with experts from intensive care medicine, with a focus on the clarity, relevance, and arrangement of the items into topics as well as the usability of the web-based questionnaire. Experts also assessed content validity (ie, whether all aspects of the topic were accurately covered by the questionnaire) and clinical validity (ie, whether the questionnaire measured the intended research topic). The final questionnaire (Multimedia Appendix 1) contained 36 items grouped into 8 topics:

- ICU staff experience with the current patient monitoring system
- Aspects of patient monitoring that disturb patient care
- Improvements for future patient monitoring
- Suggestions for remote patient monitoring display devices
- Use cases for remote patient monitoring
- Use cases for CDSS based on AI
- Aspects that promote the usage of CDSS based on AI
- Attitude of ICU staff toward novel digital technology

Additionally, respondents indicated their age group, profession, and technical affinity. For the latter, we used the Affinity for Technology Interaction Short (ATI-S) scale [11] and reduced the options from a 6-point scale to a 5-point Likert-type scale due to usability issues. Other items in the questionnaire focused on alarm management, which was the subject of another study and is not reported here.

### Data Collection

Data collection took place over a period of 2 months (November 2019 to January 2020) on an invitation basis. The sampling frame was defined as the 270 nurses and physicians working in the 4 ICUs the day before data collection began; in total, there were 177 nurses and 93 physicians. An email containing a detailed description of the study and the web address of the survey was sent to them. Study data were collected and managed using REDCap electronic data capture tools hosted at Charité – Universitätsmedizin Berlin [12,13].

To increase the survey response rate, participants were offered the opportunity to take part in a raffle to win a €50 (US $56.04) voucher for a train ticket after survey participation. Additionally, 2 reminder emails were sent to all participants 2 and 5 weeks after the initial email was sent. Finally, small handouts with a brief description of the study, the URL for the questionnaire, and a QR (quick response) code were given to ICU staff on site.

### Data Analysis

We cleaned and analyzed the data with R (R Foundation for Statistical Computing) in combination with the packages tidyverse, psych, and sjPlots [14-17]. Inferential calculations were performed with the infer package [18]. For each of the 36 five-point items, we calculated the medians and their 95% bootstrap CIs by deploying a bootstrap resampling procedure as previously described [19,20]. For the bootstrap sampling distribution, we created 15,000 bootstrap samples per item. An item median was considered statistically significant when the 95% bootstrap confidence intervals of the median did not include 3, which indicates the response “Undecided.” To compare the distributions of item responses of physicians and nurses, we used chi-square tests. Here, a two-tailed *P* value <.05 was considered statistically significant.

## Results

### Overview

This survey study is based on a questionnaire with 36 items regarding patient monitoring in the ICU, addressed to ICU staff. The actual response rate was 39.6% (107/270); however, only 86 responses from 62 nurses and 24 physicians were analyzable due to missing data. The ratio of male to female respondents was almost equal (42 men, 41 women, 3 not specified). The largest age categories were represented by participants aged 25 to 34 years (n=32, 37%) and those aged 35 to 44 years (n=28, 33%). Self-reported technical affinity (ATI-S) was rated with a mean of 3.4 (SD 0.88) and a median of 3.5 (range 2.9-4.1) on the 5-point Likert-type scale, with a Cronbach of 0.83 (95% CI 0.76-0.89).

**Figure 1:**
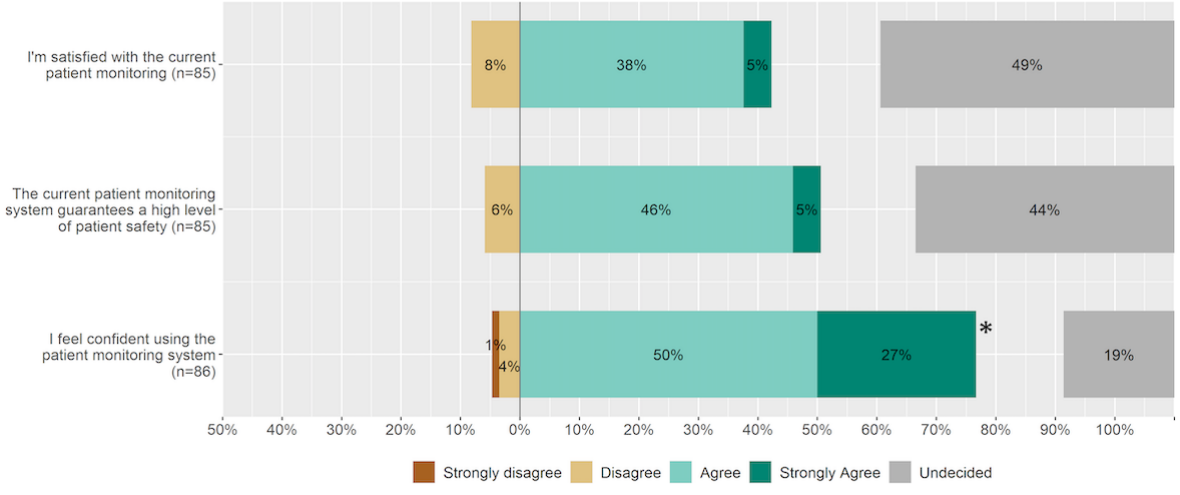
ICU staff experience with current patient monitoring. An asterisk indicates statistical significance. ICU: intensive care unit.

**Figure 2:**
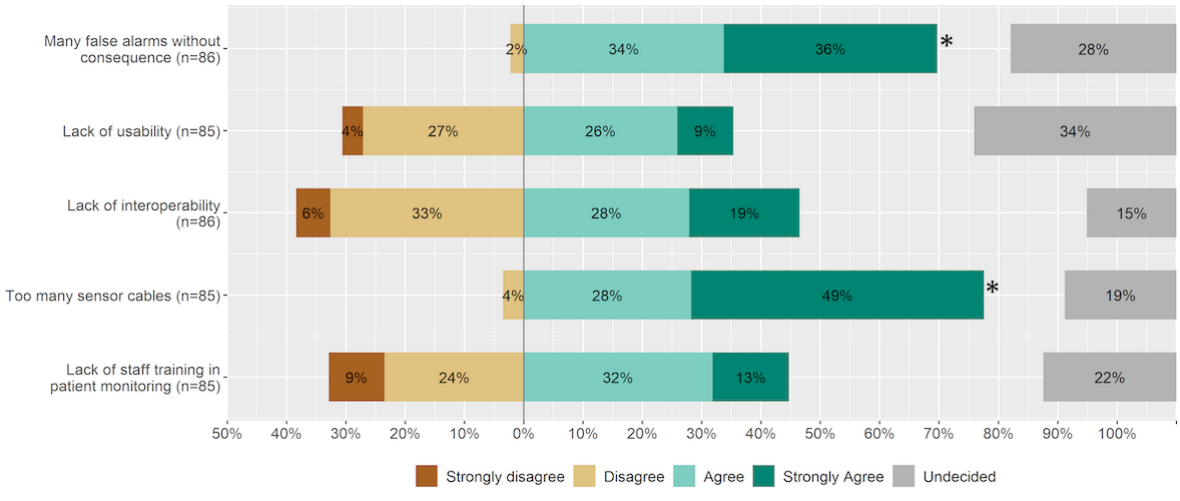
Aspects of patient monitoring disturbing patient care in the ICU. An asterisk indicates statistical significance. ICU: intensive care unit.

**Figure 3:**
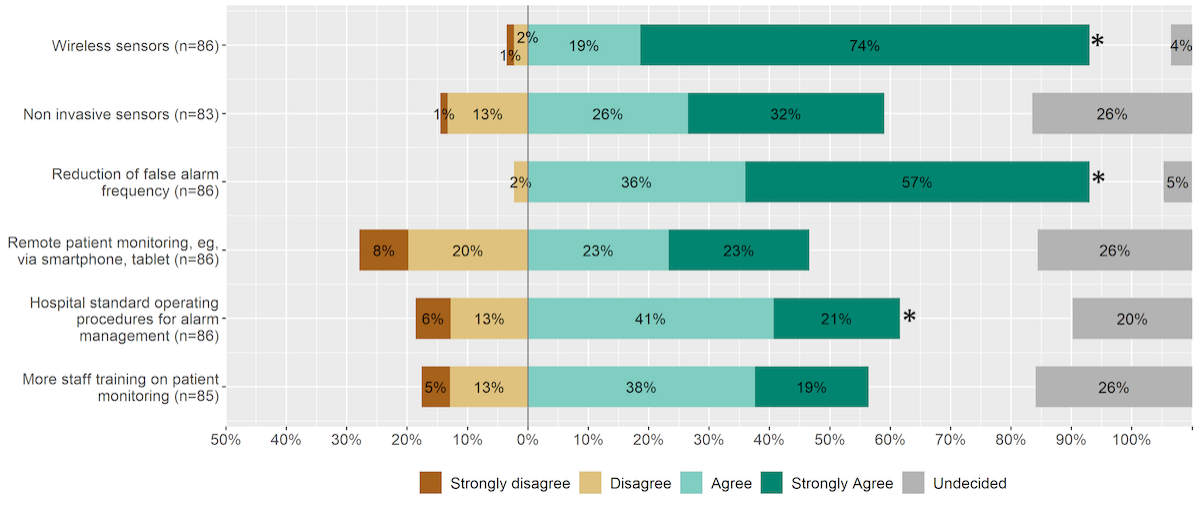
Improvements for future patient monitoring in the ICU. An asterisk indicates statistical significance. ICU: intensive care unit.

**Figure 4:**
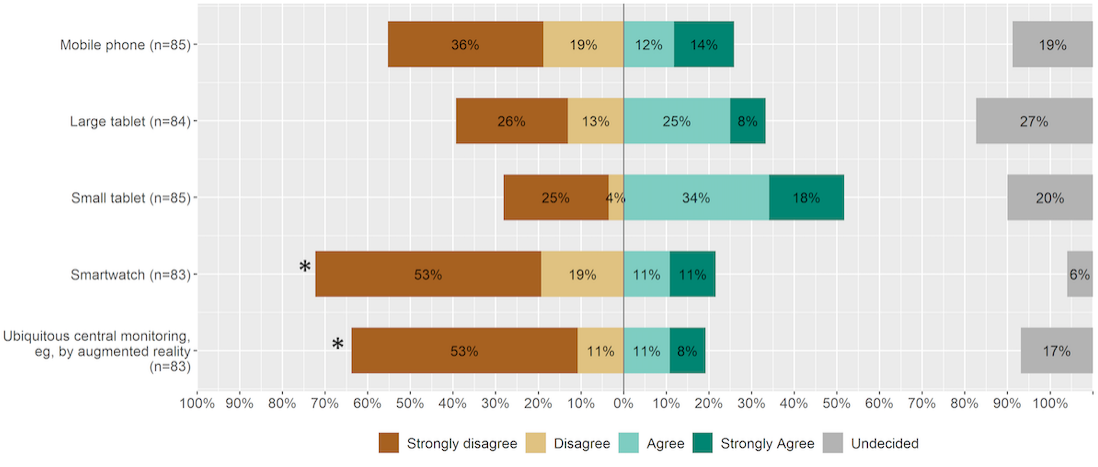
Suggestions for remote patient monitoring display devices in intensive care medicine for usage on hospital premises. An asterisk indicates statistical significance.

**Figure 5:**
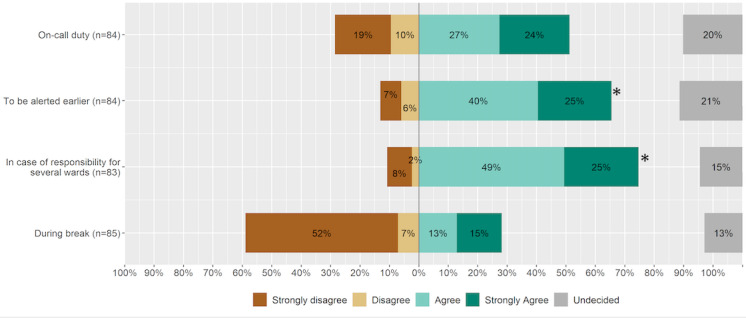
Use cases for remote patient monitoring on hospital premises for intensive care medicine. An asterisk indicates statistical significance.

**Figure 6:**
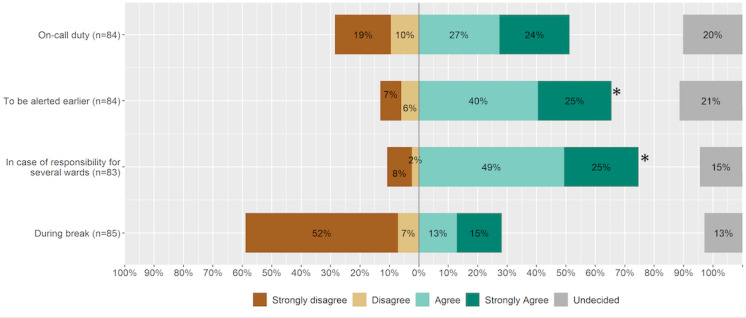
Use cases for clinical decision support systems based on artificial intelligence in the ICU. An asterisk indicates statistical significance. ICU: intensive care unit.

**Figure 7:**
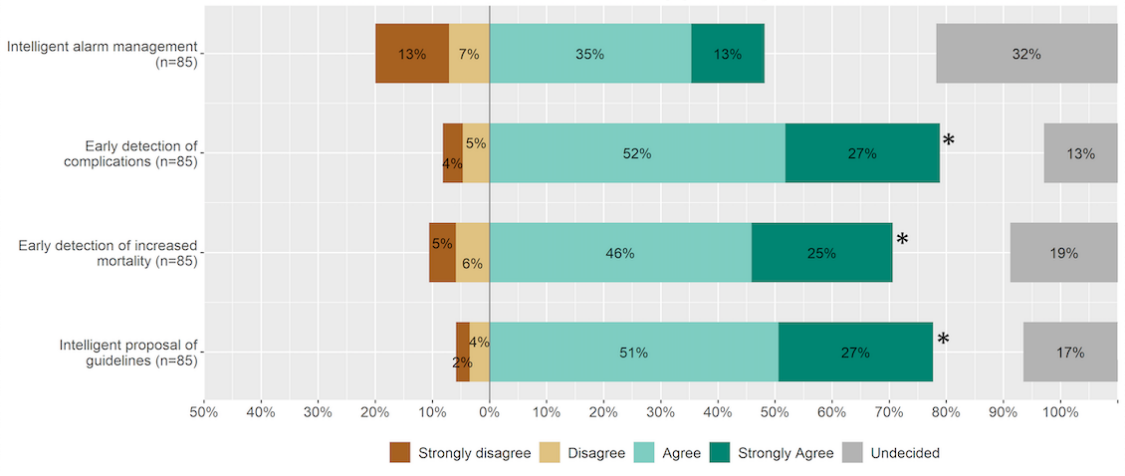
Aspects that promote the usage of clinical decision support systems based on artificial intelligence in the ICU. An asterisk indicates statistical significance. ICU: intensive care unit.

**Figure 8:**
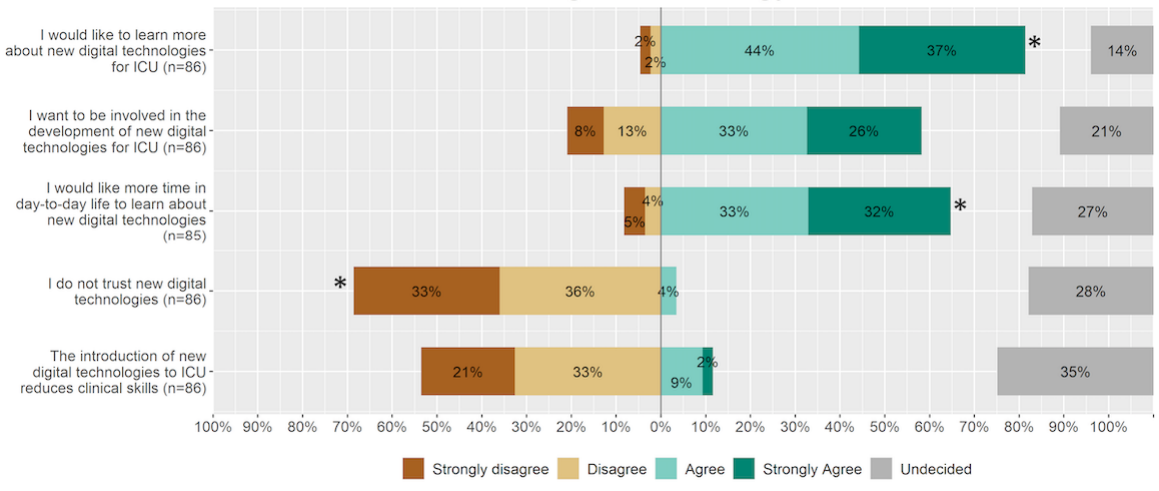
Attitude of ICU staff towards novel digital technology. An asterisk indicates statistical significance. ICU: intensive care unit.

The questionnaire results are presented as grouped Likert plots (Figures 1-8) [16], where one group represents one topic. An item median was considered statistically significant (items marked with an asterisk) when the 95% bootstrap CI of the median did not include 3, which indicates the response “Undecided” (Multimedia Appendix 2 shows item medians and bootstrap CIs). To improve readability, and in contrast to the questionnaire, the answer option “Undecided” is presented on the far right. Multimedia Appendix 3 contains the raw data, and Multimedia Appendix 4 shows the distribution of item responses of physicians and nurses.

### Current Patient Monitoring

#### ICU Staff Experience

Most of the ICU staff who took part in the online survey were satisfied with the current patient monitoring system and felt that it ensured high patient safety, even though the median responses did not differ significantly from the option “Undecided” (Figure 1). The majority stated feeling confident in using the patient monitoring system (n=66, 77% chose “Strongly agree” or “Agree”).

#### Aspects Disturbing Patient Care

The majority of respondents indicated that the patient monitoring system’s high rate of false-positive alarms (n=60, 70% chose “Strongly agree” or “Agree”) and high number of sensor cables (n=66, 77% indicated “Strongly agree” or “Agree”) interrupted patient care. The opinions about detrimental effects elicited by a lack of interoperability, lack of staff training, and low usability of the patient monitoring system were split (Figure 2).

### Suggestions for Future Patient Monitoring

#### Improvements for Future Patient Monitoring

For future patient monitoring, almost all of the ICU staff surveyed requested wireless sensors (n=80, 93% chose “Strongly agree” or “Agree”) and a reduction in false-positive alarms (n=80, 93% chose “Strongly agree” or “Agree”). False-positive alarms may occur due to measurement errors, artifacts, or incorrect settings (Figure 3). Furthermore, respondents wanted a hospital standard operating procedure (SOP) for alarm management (n=53, 62% chose “Strongly agree” or “Agree”). The median responses for the items “Noninvasive sensors,” “Remote patient monitoring,” and “More staff training on patient monitoring” did not significantly differ from the option “Undecided.”

#### Display Devices and Use Cases for Remote Patient Monitoring

According to the survey results, none of the proposed display devices were desired by ICU staff (Figure 4). The use of smartwatches or augmented reality (AR) glasses in the ICU was rejected by 72% (n=60) and 64% (n=53) of respondents, respectively (those who chose “Strongly disagree” or “Disagree”). With regard to the use of mobile phones for remote patient monitoring, nurses strongly rejected it, while physicians had a neutral attitude toward it.

The majority of respondents would appreciate a remote patient monitoring system in an intensive care setting in case they wanted to be alerted earlier (n=55, 65% indicated “Strongly agree” or “Agree”) or were responsible for multiple wards (n=62, 74% chose “Strongly agree” or “Agree”; Figure 5). Although not statistically significant, most respondents preferred a remote patient monitoring device for on-call duty, but did not find it useful while taking breaks.

#### CDSS

In the future, survey respondents would use a CDSS in the ICU that predicts complications (n=67, 79% chose “Strongly agree” or “Agree”) or the risk of mortality of patients (n=60, 71% indicated “Strongly agree” or “Agree”) as that intelligently proposes guidelines for therapy and diagnostics (n=66, 78% chose “Strongly agree” or “Agree”; Figure 6). Respondents were inclined to use it for alarm management. Physicians had fewer reservations in using a CDSS with intelligent alarm management than nurses.

Among the factors that users found essential for the use of CDSS, high interoperability (n=79, 93% chose “Strongly agree” or “Agree”) and high usability (n=78, 93% indicated “Strongly agree” or “Agree”) were deemed most essential. These were followed by the offer of regular staff training with the technology (n=75, 90% chose “Strongly agree” or “Agree”) and high transparency of the system (n=66, 78% indicated “Strongly agree” or “Agree”; Figure 7). Most physicians and nurses agreed that regular support (eg, training and workshops) promotes the use of CDSS; more physicians chose “Strongly agree,” while more nurses chose “Agree.”

#### Attitude Toward Novel Technology

Overall, survey respondents were open-minded toward novel technology; among the respondents, 81% (n=70) wanted to know more and 65% (n=55) needed more time to learn about it (“Strongly agree” or “Agree”; Figure 8). The majority (n=59, 69%) disagreed or strongly disagreed on the item “I do not trust new digital technology.” Although not statistically significant, 50 respondents (59%) wanted to be involved in the product development of novel digital technologies.

## Discussion

### Principal Findings

This survey study of ICU staff provides a substantial understanding of the needs and expectations of patient monitoring systems in intensive care medicine from the user’s perspective (Textbox 1). Although respondents were confident in using the current patient monitoring system, the high rate of false alarms and the numerous sensor cables were found to potentially interrupt patient care. ICU staff demanded wireless sensors, fewer false alarms, and a hospital SOP for alarm management. Notably, the median replies on display devices for remote patient monitoring did not differ significantly from the option “Undecided*,”* except for the items “Smartwatch” and “Ubiquitous monitoring, eg, through AR,” which were both declined. Remote patient monitoring was classified useful for earlier alerts or when responsible for several ICUs. Respondents would use a CDSS based on AI to predict complications, detect increased risk of mortality, and propose guidelines. High transparency, high interoperability, high usability, and regular staff training were all aspects that would promote its usage. Regarding digital literacy, ICU staff was eager to learn more about digital technology and spend more time with it.

**The five most anticipated improvements for patient monitoring by intensive care unit staff.**

- Reduction of false alarms
- Implementation of hospital alarm standard operating procedures
- Introduction of wireless sensors
- Introduction of a clinical decision support system based on artificial intelligence
- Enhancement of staff members’ digital literacy

### Lessons Learned From Today’s Patient Monitoring

Notably, we have not observed a proactive call to pioneer new technologies and integrate their respective digital gadgets (eg, smartwatch and AR) into clinical care. Rather, ICU staff looked forward to improvements in the functionality of existing technologies. In line with previous publications, respondents reported that the high rate of false alarms interrupted patient care and demanded a hospital SOP for alarm management [21]. In several studies, implementation of such an alarm management SOP reduced the alarm rate significantly [21,22]. Further temporal analysis of the alarm frequencies per sensor as previously described [23] may find causes for the high rate of false alarms.

It has been reported that cable entanglement is a problem in not only ICUs, but also other places where patients are monitored, such as in operating rooms [24]. Wireless sensors for monitoring vital signs have been tested and implemented several times on stepdown units [6,25]. In many cases, technical requirement analysis (eg, Bluetooth connectivity and interference with other medical devices) was conducted more than a decade ago [26,27]. However, implementation into intensive care routines is still in its infancy [28]. Reasons for this may be the costs associated with developing novel wireless sensors for a high-reliability environment such as the ICU, and technical challenges associated with the need to recharge sensors regularly. In the meantime, cord wraps may facilitate patient transfer with patient monitoring [29].

### Remote Patient Monitoring in Intensive Care Medicine

Remote patient monitoring enables clinicians to collect health data via vital sign sensors from patients at location A and electronically transfer this information to location B, where specialists access the data and give health care providers at location A recommendations for managing their patients [4]. Although this is well established in the outpatient sector between the patient's home and the physician [30], the question remains whether this can be supportive to working conditions and patient care in the ICU without a telemedicine context.

Contrary to our preceding qualitative study results, opinions regarding the need for remote patient monitoring in the ICU were divided [8]. There are several industry providers that allow ICU patients to be monitored remotely from anywhere on the premises of the hospital [31-33]. However, scientific evidence of the utility of these devices (eg, for increasing patient safety) seems to be missing. For now, we can summarize that the advantages of on-premise remote patient monitoring for intensive care medicine have to be further quantified by measures such as the reduction of alarms, and improved patient outcomes such as a reduction in patient length of stay.

### CDSS in Intensive Care Medicine

As the amount of data as well as the complexity of diseases and treatment of ICU patients are increasing, it seems reasonable to augment the abilities of ICU staff by implementing CDSS based on AI in the ICU. Our results indicate that most of the topics proposed (eg, prediction of mortality, prediction of complications, or proposal of guidelines) were seen as potential use cases for CDSS by ICU staff. For these and several other instances, algorithms already exist that could be adjusted for real-time data [34].

On the path toward implementing CDSS based on AI in intensive care medicine, several barriers have to be overcome [35]. With the introduction of the electronic health record and PDMS in the ICU, the first step has been taken to establish the technical infrastructure, but these systems need to be optimized in interoperability and data quality to act as the basis for complex machine learning processes. To utilize the power of AI as soon as possible, hospital providers should focus on developing data science departments, and introduce standards in implementing novel CDSS tools to rapidly address technical, legal, ethical, and privacy issues.

### Transdisciplinary Research and Development

Clinical teams in ICUs are used to working closely together in multidisciplinary teams. This could be advantageous when adding further professions to the team for transdisciplinary research and the development of medical devices for intensive care medicine [36]. Our survey results show that ICU staff members are open to learning more about technology and are even willing to support product development in some cases. Thus, a clinical data scientist with formal medical training could be part of the ICU team as well as the product development team alongside engineers from a medical manufacturer [22,37]. This transdisciplinary approach should be piloted in further studies, to assess the effects on mutual exchange and innovation potential.

As much as the transdisciplinary approach is supported, blunt confidence in user feedback will mainly improve existing devices, as our study prominently indicates, which does not necessarily foster the discovery of disruptive technologies [38], such as avatar-based patient monitoring [39,40] or smart glasses [41]. More than cooperation, transdisciplinarity refers to the development of common theories, mutual observation, and search for challenges and needs [42]. Hackathons (weekend innovation events) are an excellent playground for transdisciplinary work, and participation should be encouraged and remunerated by medical manufacturers and hospital providers [43].

### Limitations

With this survey study among ICU staff, we identified the most anticipated improvements for patient monitoring in the ICU from the user perspective. However, several limitations apply to this study. It is important to note that the developed questionnaire did not include questions of established reliability or validity; the data were collected at a single hospital in Germany; the number of participating physicians was small, making statements about group comparisons susceptible to coincidence; and the response rate was moderate. Due to the online collection of data, the participation of ICU staff with less technical affinity may have been reduced. Further studies including a sample size calculation and randomized sample collection would reduce the risk of bias.

Whether the findings (eg, introducing wireless patient monitoring sensors) actually lead to an improvement in working conditions and patients’ quality of life or quality of care in the ICU can only be ascertained by further studies. Finally, a bias due to the deployment of the Vital Sync virtual patient monitoring platform in 1 of the 4 ICUs cannot be ruled out with certainty.

### Conclusion

This survey study among ICU staff revealed anticipated key improvements for patient monitoring in intensive care medicine from the user perspective. We did not observe a proactive call to pioneer new technologies and integrate their respective digital gadgets (eg, smartwatch and AR) into clinical routine. Instead, ICU staff looked forward to improvements in the functionality of existing technologies. Particularly, hospital providers and medical device manufacturers should focus on reducing false alarms, implementing hospital alarm SOPs, introducing wireless sensors, preparing for CDSS based on AI, and enhancing the digital literacy of ICU staff. In the medium term, our results may contribute to the user-centered transfer of digital technologies into practice to alleviate challenges in intensive care medicine, such as those recently caused by COVID-19.

## Appendix

Multimedia Appendix 1 Final questionnaire.

Multimedia Appendix 2 Survey item medians and bootstrap CIs.

Multimedia Appendix 3 Survey raw data.

Multimedia Appendix 4 Distribution of item responses of physicians and nurses.

## Abbreviations

AI: artificial intelligence
AR: augmented reality
ATI: Affinity for Technological Interaction
CDSS: clinical decision support system
COVID-19: coronavirus disease
ICU: intensive care unit
PDMS: patient data management system
QR: quick response
SOP: standard operating procedure
