# Supplementary figures and images for: Improvements in Patient Monitoring in the Intensive Care Unit: Survey Study
Source: J Med Internet Res. 2020 Jun 19;22(6):e19091. doi: 10.2196/19091 (PMC7307326; doi:10.2196/19091)

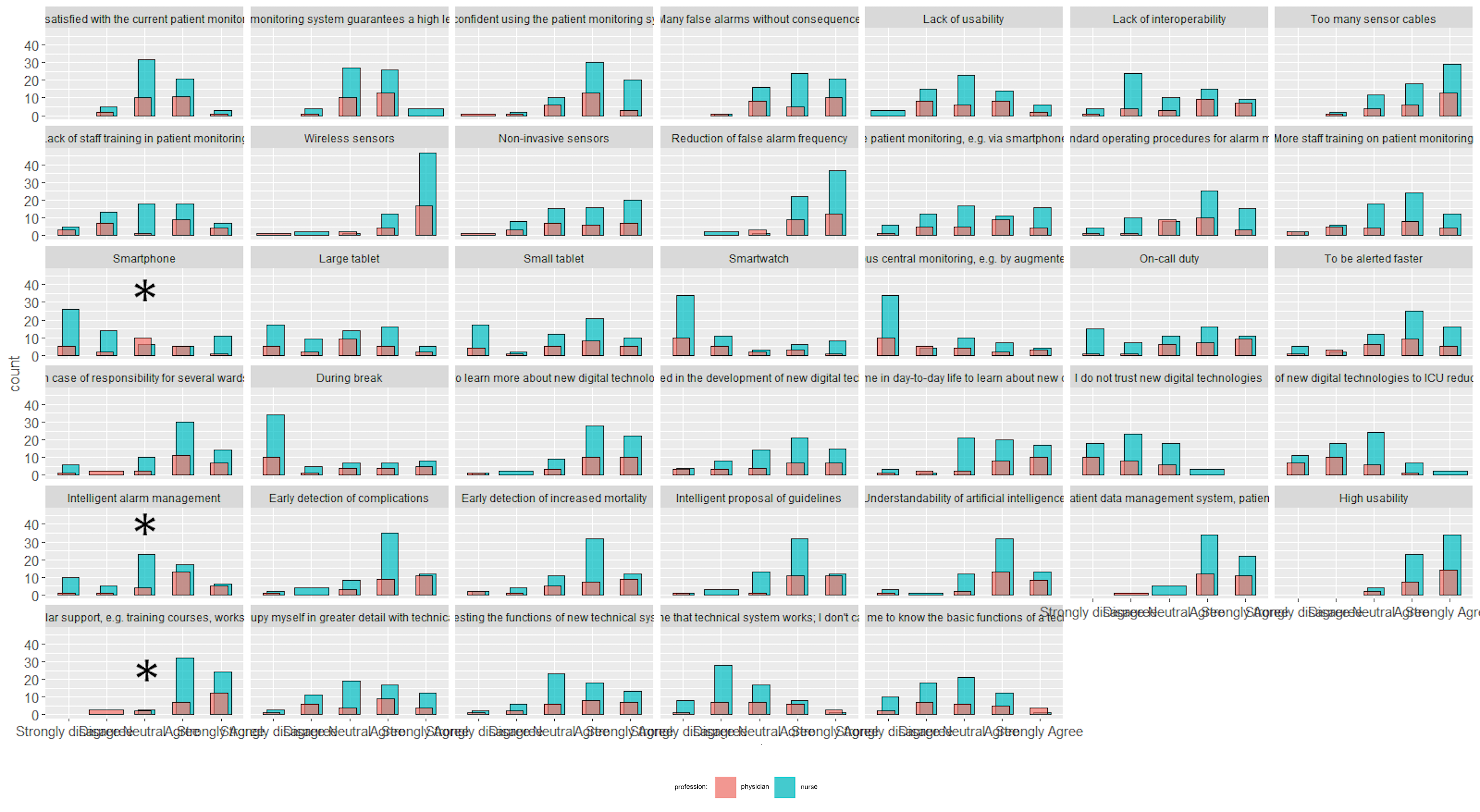

Supplement: Multimedia Appendix 4 [file jmir_v22i6e19091_app4.png]
